# Meat Characteristics, Expression of Myosin Heavy Chain and Metabolism-Related Genes in Thai Native Pigs

**DOI:** 10.3390/foods13101502

**Published:** 2024-05-13

**Authors:** Chanporn Chaosap, Kamon Chaweewan, Kazeem D. Adeyemi, Netanong Phonkate, Ronachai Sitthigripong

**Affiliations:** 1 Department of Agricultural Education, Faculty of Industrial Education and Technology, King Mongkut’s Institute of Technology Ladkrabang, Bangkok 10520, Thailand; 2 Bureau of Animal Husbandry and Genetic Improvement, Department of Livestock Development, Muang District, Pathum Thani 12000, Thailand; krawan2001@gmail.com; 3 Department of Animal Production, Faculty of Agriculture, University of Ilorin, Ilorin PMB 1515, Nigeria; kazyadeyemi@gmail.com; 4 Department of Animal Technology and Fishery, Faculty of Agricultural Technology, King Mongkut’s Institute of Technology Ladkrabang, Bangkok 10520, Thailand; netanong61@gmail.com (N.P.); ksronach@kmitl.ac.th (R.S.)

**Keywords:** muscle fiber type, inosine monophosphate, sarcomere length, shear force, glycogen, fatty acid

## Abstract

This study investigated the meat quality, expression of myosin heavy chain (MyHC) and metabolism-related genes, ribonucleotides and fatty acids in *Longissimus thoracis* of Thai native pigs (TNPs) from different geographical regions (GR). Forty-one 9–10-month-old castrated TNPs (BW 60 kg), consisting of 18, 11 and 12 pigs from Northern (NT), Southern (ST) and Northeastern (NE) regions, respectively, were slaughtered. GR did not affect (*p* > 0.05) the expression of MyHC, phosphoglycerate mutase 1, cytosolic glycerol-3-phosphate dehydrogenase, triosephosphate isomerase 1 and adipocyte fatty acid binding protein genes. The trend of MyHC was MyHC IIx > MyHC IIb > MyHC IIa > MyHC I. The NT loin had higher (*p* < 0.05) glycogen, C18:2n6, C20:4n6 and cooking loss, lower inosine, inosine monophosphate and hypoxanthine and a shorter sarcomere length than the ST and NE loins. The ST loin had a lower (*p* < 0.05) a* compared to other loins. Principal component analysis established significant relationships between the TNP and specific meat quality traits. This finding suggests that GR affected the meat quality, ribonucleotides and selected fatty acids in TNPs. These results provide relevant information that can be used to optimize the use of Thai native pork.

## 1. Introduction

Native pigs play a significant role in farming systems and rural economies owing to their ability to withstand harsh climatic conditions, their high disease resistance, them requiring minimal inputs and their ability to easily be integrated into smallholder farming systems [1,2,3]. Native pigs are known for their distinctive taste, texture and flavor [2,4]. Promoting native pigs supports local food production and improves the availability of high-quality, nutritious meat in local markets [2,4].

In Thailand, native pig rearing is distributed in three different regions: the north, northeast and south [5,6]. An analysis of microsatellites reveals a mosaic of genetic variation among these indigenous pig populations, indicating their unique geographic origins [6]. As of 2023, there were 367,993 native Thai pigs out of the total of 11,172,465 pigs in Thailand [7]. Pork production in Thailand reached 890.74 metric tons in 2022, a marginal decline from the year before, with Thai native pork accounting for 3.29% [7]. In recent times, there is an increasing demand and preference for native pork among consumers owing to its perceived healthiness and quality [2,4]. However, unlike other native pigs whose meat characteristics have been examined [8,9,10,11], little is known about the meat properties of TNPs. Exploring the meat quality of native pigs is important for conservation, economic, cultural and culinary reasons. By understanding and enhancing the meat quality of these breeds, we can ensure their long-term sustainability and value in the market. The arrangement and distribution of muscle fibers largely determines meat quality [12,13,14]. Meat’s texture, tenderness, juiciness and overall eating experience can all be influenced by the size, length and arrangement of its muscle fibers [8,9,15]. Muscle fiber types in several native pigs have been investigated [8,9,10,11]. However, muscle fibers in TNPs and their relationship with meat quality have been barely studied.

The dynamic competition between muscle and fat accretion in pigs involves intricate physiological processes in which innumerable genes are up- and down-regulated and are involved in various biological processes that directly impact meat quality [16,17,18]. Myofibrillar formation and muscle development are significantly influenced by the myosin heavy chain (MyHC) genes [8,10,11]. In addition, adipocyte fatty acid binding protein (A-FABP), triose phosphate isomerase 1 (TPI−1), phosphoglycerate mutase 1 (PGAM1) and cytosolic glycerol-3-phosphate dehydrogenase (cGPD) play key roles in fat metabolism and glycolysis [16,19,20]. Currently, little is known about the expression of such genes in TNPs, and thus, the molecular mechanisms underlying variations in meat quality in TNPs are poorly understood. The breed of pigs can exert a significant impact on its fat metabolism-related genes, ultimately influencing its fat deposition, composition and overall metabolic health [20,21]. Understanding these breed-specific differences can help producers better manage and optimize the production of pork products based on desired fat content and quality. Therefore, the aim of this study was to investigate the meat quality, the expression of myosin heavy chain and fat metabolism-related genes and the ribonucleotides and fatty acids in the Longissimus muscle of native pigs from different geographical regions of Thailand.

## 2. Materials and Methods

### 2.1. Pig Husbandry and Slaughtering

Forty-one 5-month-old castrated male Thai native pigs with a mean body weight of 21.0 ± 2.9 kg, consisting of 18, 11 and 12 pigs from Northern (NT) Mae Hong Son province, Northeastern (NE) Ubon Ratchathani province and Southern (ST) Nakhon Si Thammarat province, respectively, were reared under the same conditions and fed commercial fattening pig diets at the Swine Research and Development Center, Department of Livestock Development, Thailand. Once they reached a body weight of 60 kg at about 9–10 months, the pigs were fasted for 18 h, with free access to water. They were electrically stunned, slaughtered, skinned and eviscerated, and the carcasses were split lengthwise and chilled at 1–4 °C.

### 2.2. Muscle Sampling

Approximately 100 g of *Longissimus thoracis* (LT) at rib 8-12 was collected from 18 NT pigs, 11 NE pigs and 12 ST pigs within 1 h of postmortem, snap-frozen in liquid nitrogen and stored at −80 °C until the analysis of gene expression and glycogen. At 24 h postmortem, the carcasses were dissected and the LT muscle sample was taken from the left side of each carcass before being divided into two 1.5 cm-thick slices and one 3 cm-thick slice. The first 1.5 cm-thick slice was vacuum-packed and stored at −80 °C for ribonucleotide and fatty acid analysis. The second 1.5 cm-thick slice was used to determine the muscle fiber diameter, sarcomere length and proximate analysis. The meat color of the 3 cm-thick slice was measured, stored individually and vacuum-packed in a barrier bag at −20 °C until the meat quality analysis.

### 2.3. Gene Expression

#### 2.3.1. RNA Extraction and cDNA Synthesis

After the extraction of the total RNA from each sample, a deoxyribonuclease was used to remove genomic DNA (gDNA) contaminants. Subsequently, cDNA was synthesized using random primers, nuclease-free water and a transcription mixture (Revert Aid First Strand cDNA Synthesis Kit, Thermo Scientific, Waltham, MA, USA). The primers used in the current study are listed in Table 1.

#### 2.3.2. Quantitative Real-Time PCR

First-strand cDNA from the samples was diluted 1:5, and then a cDNA pool was created for each sample and a dilution series was generated for use as a standard curve. Individual samples were diluted 1:4 for gene expression analysis. A total of 3.5 µL of cDNA, 0.4 µL of forward and reverse primers and 5 µL of SYBR Green Universal PCR Master Mix (SensiFastTM SYBR, BIOLINE, London, UK) formed the reaction mixture, which was duplicated on a 96-well plate on a Bio-Rad CFX96 system (Bio-Rad, Hercules, CA, USA): 95 °C for 2 min, followed by 40 cycles of 95 °C for 5 s and 55 °C for 15 s; fluorescence was monitored in real time. To determine the specificity of the interaction, the melting curve was evaluated after the PCR procedures using CFX Manager™ software Version 3.1, (Bio-Rad, Hercules, CA, USA). The average mRNA expression of the PGAM−1, cGPD, TPI−1 and A-FABP genes was normalized relative to the expression of the reference gene glyceraldehyde-3-phosphate dehydrogenase (GAPDH).

Relative standard curves were generated for each MyHC isoform primer set to calculate PCR efficiency. The Cq values (*y*-axis) were plotted against the log10 ng equivalent RNA (*x*-axis), and PCR reaction efficiencies (E) were calculated from the standard curve as 10^(−1/slope)^ − 1 [23]. An average Cq value was obtained for each primer set of each sample, and this was used to calculate the relative expression ratio (rER).

$$rER=\frac{{\left[1+E(MyHC\;target\;gene)\right]}^{–Cq(MyHC\;target\;gene)}}{{\left[1+E(MyHC\;control\;gene)\right]}^{–Cq(MyHC\;control\;gene)}}$$

#### 2.3.3. Chemical Composition and Glycogen Analysis

Meat samples were analyzed for dry matter and ether extract using AOAC [24] methods. Muscle glycogen was measured according to Dreiling et al. [25].

#### 2.3.4. Ribonucleotide Analysis

One gram of the crushed sample was homogenized in 6 mL of cold 0.6 M perchloric acid; then, 0.8 M KOH and KH_2_PO_4_ buffer were used to neutralize the homogenate and adjust the pH to 7.0. The mixed sample was centrifuged at 10,000× *g* at 4 °C. The supernatants were used to determine the concentration of inosine monophosphate (IMP), inosine, hypoxanthine and guanosine monophosphate (GMP) using an HPLC (Chromaster, Hitachi, Tokyo, Japan) with a UV detector (Acetonitrile:KH_2_PO_4_, 70:30 buffer was the elution phase and TSK Gel Amide-80 column (Tosoh, Tokyo, Japan) was the stationary phase). The ribonucleotide content was determined using an external standard curve [26].

#### 2.3.5. Meat Quality Analysis

The pH was measured in duplicate at 45 min and 24 h postmortem directly on each LT muscle, with a pH meter equipped with a spear tip glass electrode (SevenGo, Mettler-Toledo International Inc., Greifensee, Switzerland). The pH was calibrated prior to taking readings by dipping the pH probe into buffers (pH 4 and 7).

A portable spectrophotometer (MiniScan EZ, illuminance D65, 10° observer, Hunter Associates Laboratory Inc, Reston, VA, USA) with an aperture of 2.54 cm in diameter was used to measure color coordinates CIE L*, a* and b* in triplicate at different locations of each muscle sample after 45 min of air exposure at 25 °C.

After weighing, each 3 cm-thick slice was sealed in a high-density polyethylene bag and cooked in an 80 °C water bath until the core temperature reached 70 °C. The difference in weight between the precooked and cooked samples was evaluated and expressed as a percentage of the weight before cooking to measure cooking loss.

For Warner Bratzler Shear Force (WBSF) measurements, each cooked sample was cut into eight 1.3 × 1.3 × 3 cm^3^ cubes, perpendicular to the muscle fiber. Each cube was sheared perpendicular to the muscle fibers using a texture analyzer (EZ-SX, Shimadzu, Kyoto, Japan) with a 50 kg load cell and a crosshead speed of 50 mm/min [26].

#### 2.3.6. Sarcomere Length and Muscle Fiber Diameter Measurement

The sarcomere length was determined using the helium–neon laser diffraction method described by Cross et al. [27]. An average of thirty sarcomere lengths were measured from each sample. For muscle fiber diameter analysis, the sample was fixed in 10% formalin for 48 h before mixing with 0.9% sodium chloride solution. The mixture was then blended for 30 s. Two hundred fibers from the mixed samples were randomly selected for measurement using a 4× microscope equipped with a Dino-Eye eyepiece camera (Dino Lite 2.0, New Tapie City, Taiwan, China). The data were then processed using Dino Capture Version 2.0 software (AnMo Electronics Corporation, New Taipei City, Taiwan).

#### 2.3.7. Fatty Acids Analysis

The total lipids of the meat samples were extracted with a chloroform:methanol mixture (2:1, *v*/*v*). The extracted lipids were transmethylated into fatty acid methyl esters (FAMEs) according to AOAC methods [24]. The internal standard used during extraction was methyl nonadecanoate (C19:0). FAMEs were analyzed by gas chromatography (7890B, Agilent, Santa Clara, CA, USA) using a fused silica capillary column (100 m × 0.25 mm × 0.2 μ film thickness, SPTM-2560, Supelco, Bellfonte, PA, USA) equipped with a UV detector. The injector and detector temperatures were 240 °C and 260 °C, respectively. Carrier gas was Helium at a constant pressure of 110.32 kPa and a split ratio of 10:1. The column was operated at an initial temperature of 60 °C, followed by an increase at 20 °C/min to 170 °C, 5 °C/min to 220 °C and then 2 °C/min to 240 °C. Sample peaks were identified, and concentrations were calculated by comparison with peak areas and retention times of known FAME standards. The atherogenic index (AI) and thrombogenic index (TI) were calculated according to Ulbricht and Southgate [28] as follows:

AI = (C12:0 + 4 × C14:0 + C16:0)/(ΣMUFA + Σn6 + Σn3)

TI = (C14:0 + C16:0 + C18:0)/[(0.5 × ΣMUFA + 0.5 × Σn6 + 3 × Σn3)+(n3/n6)]

#### 2.3.8. Statistical Analysis

The data were checked for normality using the PROC UNIVARIATE procedure of SAS (SAS Institute Inc., Cary, NC, USA) and found to be normally distributed. The data were subjected to the generalized linear model procedure of SAS. A pig was the experimental unit. The experimental model included the geographical region as a fixed effect, while individual pigs were included as a random effect. The level of statistical significance was set at *p* < 0.05. Least-square means were separated using the PDIFF option. The principal component analysis (PCA) of the studied parameters (meat characteristics, ribonucleotides, fatty acids and the expression of myosin heavy chain isoforms and metabolism-related genes) was analyzed using the XLSTAT software version 2023.3.1 (Addinsoft, New York, NY, USA).

## 3. Results

### 3.1. Expression of Myosin Heavy Chain and Metabolism-Related Genes

Geographical regions had no effect (*p* > 0.05) on the MyHC composition in the LT of TNPs (Table 2). In addition, the expression of PAGM−1, cGPD, TPI−1 and A-FABP genes was not affected (*p* > 0.05) by GR (Table 2).

### 3.2. Chemical Composition, Glycogen and Ribonucleotide Contents

GR had no effect on the fat, moisture and GMP contents in the TNPs (Table 3). The NT loin had a higher (*p* < 0.05) muscle glycogen content than the ST and NE loins. The NT loin had lower (*p* < 0.05) inosine, hypoxanthine and IMP contents than the ST and NE loins (Table 3). The glycogen, hypoxanthine, inosine and IMP contents did not differ between the ST and NE loins.

### 3.3. Meat Quality Characteristics

GR had no effect (*p* > 0.05) on the pH_45_, lightness, yellowness, shear force and muscle fiber diameter in the loin of TNPs (Table 4). The muscle pH_24_ of the NE loin was higher (*p* < 0.05) than that of the NT loin. The pH_24_ of the ST loin was not different from that of the NT and NE loins. The redness of the ST loin was lower (*p* < 0.05) than that of the NT and NE loins. The cooking loss was higher (*p* < 0.05) and the sarcomere length was shorter (*p* < 0.05) in the NT loin than in the ST and NE loins.

### 3.4. Muscle Fatty Acids

The fatty acid (FA) composition of TNPs is shown in Table 5. The NT loin had a lower (*p* < 0.05) C16:0 compared to the NE loin. The concentration of C16:0 in the ST loin was similar to that of the NT and NE loins. The GR had no effect on the concentration of C14:0, C18:0, C16:1, C18:1n-9 and C18:3n-3 in TNP loins. The concentration of C18:2n6c, C20:4n6 and total polyunsaturated fatty acids (PUFA) and the P:S ratio were greater (*p* < 0.05), while total saturated fatty acids (SFA) were lower in the NT loin than in the ST and NE loins.

### 3.5. Principal Component Analysis

**Figure 1:**
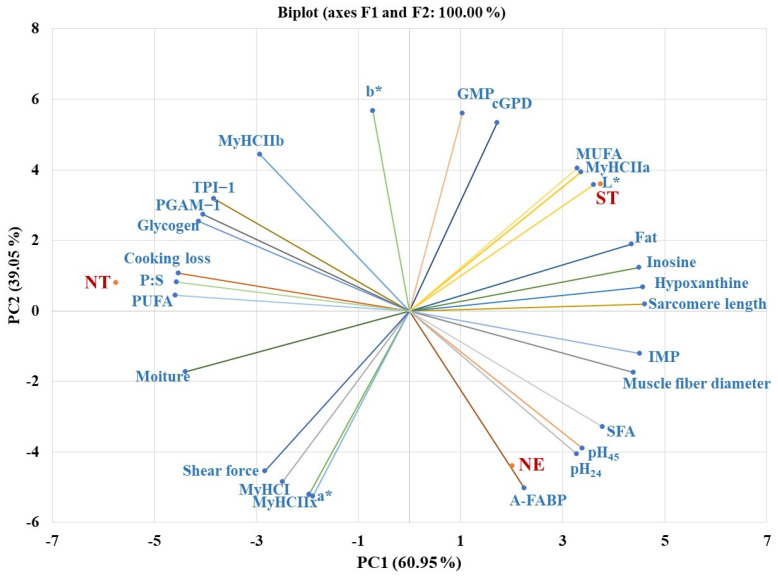
Principal component analysis biplot of Thai native pigs from different geographical regions and meat characteristics. Geographical regions: NT = Northern, ST = Southern and NE = Northeastern region. MyHC = Myosin heavy chain isoform, A-FABP = adipocyte fatty acid binding protein, TPI−1 = triose phosphate isomerase 1, PGAM−1 = phosphoglycerate mutase 1, cGPD = cytosolic glycerol-3-phosphate dehydrogenase, IMP = inosine monophosphate, GMP = guanosine monophosphate, SFA = saturated fatty acids, MUFA = monounsaturated fatty acids, PUFA = polyunsaturated fatty acids, P:S = PUFA:SFA.

Principal component analysis (PCA) was performed to evaluate the relationships between TNPs from different geographical regions and their meat characteristics (Figure 1). PC1 and PC2 together explained 100% of the total variance. The PC1 (60.95% of thr total variance) was positively loaded by the MHCIIa, %fat, pH45, pH24, L*, muscle fiber diameter, sarcomere length, hypoxanythine, inosine, IMP, SFA and MUFA but negatively loaded by the PGAM−1, TPI−1, glycogen, %moisture, cooking loss, PUFA and P:S. The PC2 (39.05% of total variance) was positively loaded with the MyHCIIb, cGPD, b* and GMP but negatively loaded with the MyHCI, MyHCIIx, A-FABP, a* and shear force. From the bi-plot, the NT loin was closely related to TPI1, PGAM−1, glycogen, moisture, cooking loss, PUFA and P:S, while the ST loin on the opposite side was closely related to the MyHCIIa, L*, MUFA, fat, inosine, hypoxanthine and sarcomere length. The NE loin was closely related to the MyHCI, MyHCIIx, A-FABP, pH45, pH24, a* and shear force.

## 4. Discussion

The muscle fiber composition is a key factor in meat quality [11,29]. GR did not affect the gene expression of MyHC isoforms in TNPs. This observation could be attributed to the uniform slaughter weight (60 kg) of the pigs, suggesting homogenous muscle and fat accretion. The slow growth rate of native pigs could potentially mask significant changes in MyHC isoforms. Similarly, the breed had no effect on the muscle fiber composition in Iberian pigs and their crosses [30]. According to Wimmers et al. [11], the muscle fiber type classified by histochemistry had a highly significant correlation with the corresponding MyHC isoforms, as evident by real-time PCR quantification of the MyHC isoform transcripts. We present, for the first time, the composition of MyHC isoforms in TNPs. Regardless of GR, the trend of MyHC isoforms was MyHC IIx > MyHC IIb > MyHC IIa > MyHC I. The MyHC composition observed herein is comparable to that of native pigs in other studies [16,31]. A notable observation in our study was that, irrespective of GR, intermediate MyHC IIx was the predominant fiber type in the LT of TNPs, as its gene expression accounted for approximately 70% of the total MyHC gene expression. This result supports previous data indicating the prevalence of MyHC IIx in the *Longissimus* muscle of native pigs such as Jinhua [31] and Meishan [16] and is in contrast to those of commercial breeds, in which MyHC IIb predominates [16,31]. An increase in glycolytic MyHC IIb increases muscle mass, while an increase in oxidative MyHC I and oxidative glycolytic MyCH IIa and MyCH IIx increases the color stability, tenderness and water holding capacity [10,11]. Thus, our results confirm the better meat quality in TNP, which is consistent with previous results in other native pigs [16,31].

Metabolic-related genes are essential for regulating the biochemical processes that determine the quality attributes of meat, including the flavor, tenderness, juiciness and texture [16,17,18]. Understanding the role of these genes in meat quality can help researchers and producers improve breeding and management practices to enhance the overall quality and value of meat products. In this study, we examined the expression of PGAM1, cGPD, TPI−1 and A-FABP genes, which play important roles in glycolysis and fat metabolism [16,21,32,33]. Since the MyHC composition and IMF content were not affected by GR, we do not expect differences in the expression of genes related to energy and fat metabolism. Therefore, the similarity in the expression of the A-FABP, cGPD, PGAM1 and TPI−1 genes suggests similar muscle and IMF accretion in the GR groups.

The relationships between the IMF content and sensory and technological properties of meat are well established [34]. The IMF largely reflects the balance between the synthesis, degradation and uptake of triglycerides [20,21]. Thus, the similar expression of genes related to fat deposition and metabolism is consistent with the similar IMF in TNPs. An intramuscular fat content of 1.5% or 2–3% is required for a pleasant eating experience or optimal eating quality, respectively [21,34]. In this study, the IMF content ranged from 3.05 to 3.56%, which is consistent with the data for the *Longissimus* muscle of various native pigs [4,35,36].

The muscle glycogen content may influence the rate and extent of postmortem pH decline [37]. The higher glycogen content in NT compared to that in ST and NE could be due to the slightly higher, although not significant, MyHC IIb gene expression in the NT loin. MyHC IIb is rich in glycogen and has higher glycolytic enzyme activity [37].

Nucleotides contribute significantly to the meat flavor [38]. The postmortem degradation of ribonucleotides such as adenosine triphosphate (ATP) and guanosine triphosphate (GTP) produces flavor-related compounds such as GMP and IMP, which are further degraded to form inosine and hypoxanthine [38,39]. The IMP and GMP enhance the umami flavor, hypoxanthine has a bitter taste and while inosine has no taste [38,39]. As noted in previous studies [36,38], IMP was the major nucleotide in the LT of TNPs. The NT loin had lower contents of inosine, hypoxanthine and IMP than the ST and NE loins. This could be due to the low pH of the NT loin, which accelerates IMP dephosphorylation [40]. In addition, since inosine, hypoxanthine and IMP are water-soluble [38], it is possible that the lower pH induces a greater purge loss, which could have resulted in the loss of ribonucleotides. Our results are consistent with those of Tikk et al. [38], in which a lower pH reduced the concentration of IMP and hypoxanthine in fresh pork. The greater IMP in the ST and NE loins may indicate that they are likely to produce more umami flavor than the NT loin.

The values of the shear force (5.05–5.74 kg) and color coordinates in this study are comparable to those reported for the *Longissimus* muscle of various native pigs [35,36]. In addition, the range of cooking loss (14.86–20.79%) obtained in this study is comparable to the 10–27% obtained in different breeds of pigs [41]. The lower pH_24_ in the NT loin could be due to the higher glycogen content. In postmortem muscle, glycogen and other substrates are converted to lactic acid by postmortem glycolysis. The accumulation of lactate leads to a decrease in the muscle pH [37]. The lesser redness of the ST loins compared to that of the NT and NE loins may be due to differences in the amount and oxidation state of myoglobin [42]. The greater cooking loss in the NT loin may be due to the lower muscle pH. At a low muscle pH, which is close to the isoelectric point of myofibrillar proteins, especially myosin, the amount of water attracted to protein structures in the myofibril would decrease [43]. The reduced sarcomere length observed in the NT loin could be due to a lower pH, as the decreased muscle pH postmortem leads to an increase in the free calcium level in the sarcoplasm, consequently enhancing the interaction between actin and myosin [44]. Similar to our findings, breed- and strain-related differences in pH, color coordinates and cooking loss were found in native pigs [4,36].

Muscle fatty acids play a vital role in the eating and technological qualities and healthiness of meat [21]. Regardless of GR, the total content of MUFA, SFA and PUFA in the LT of TNPs ranges from 50 to 53, from 29 to 36 and from 12 to 21%, respectively. A similar trend of FA distribution has been documented in various native pigs [4,45,46]. In pigs, PUFA are obtained from the diet, while MUFA can be supplied from the diet or synthesized de novo [21,47]. The higher MUFA and SFA contents in the TNPs were consistent with the greater ability of native pigs to synthesize and desaturate lipids, which consequently lowers the PUFA content due to dilution effects [21,47]. The lower C16:0 and total SFA in the NT loin may be due to its greater C18:2n-6, C4n-6 and total PUFA contents because PUFA can suppress the activities of lipogenic enzymes that are responsible for the endogenous synthesis of C16:0 and other SFA [48,49]. The higher C18:2n6 content in the NT loin may indicate that the pigs have a higher capacity to deposit this FA compared to other pigs. The higher C20:4n6 content in NT was expected because C20:4n6 is a long-chain metabolite of C18:2n6 [47]. Our findings indicate that in spite of the similar IMF content in the NT, ST and NE loins, some differences in muscle fatty acids may occur. This observation is in agreement with that of Garrido et al. [46], who found differences in the FA composition of Lampiño, Torbiscal and Retinto pigs despite similar IMF contents. The higher PUFA content in the NT loin suggests that it may probably be more susceptible to lipid oxidation compared to the ST and NE loins.

The PUFA/SFA and n-6/n-3 ratios, TI and AI are good markers of the healthiness of fat in foods. The recommended PUFA/SFA is ≥0.4 [50,51]. This target was met in only the NT loin, whose PUFA/SFA was 0.75, compared to that of 0.34 in ST and NE loins. This was probably due to the greater PUFA and lower SFA in the NT loin compared with the NE and ST loins. A ratio of <4 is recommended for n-6/n-3 [50,51]. This target was not met in this study, regardless of GR. Likewise, the n-6/n-3 of the LT of native prestice black-pied [52], Ibérico [41], Alentejana [45] and Iberian [30,46] pigs was >4. The AI and TI in the NT loin were lower than those of the NE and ST loins as a result of the decrease in C16:0 and the increase in the total PUFA. Nevertheless, the range of AI and TI observed in this study was in line with those observed in several native pig breeds [41,45,46].

The PCA result shows that the parameters related to chemical composition and meta-related gene expression are consistent with the PC1 axis. Muscles that have a high expression of PGAM−1 and TPI−1 genes had a positive relationship with a high glycogen content, PUFA, PUFA:SFA, moisture and a high cooking loss but a negative relationship with the SFA, %fat, inosine, hypoxanthine, IMP, L*, pH_45_, pH_24_, sarcomere length and muscle fiber size. These attributes are typical of glycolytic muscles. Both PGAM−1 and TPI−1 play vital roles in postmortem glycolysis [32,33]. In the presence of a high glycogen content and a larger proportion of inorganic phosphate that promotes the conversion of glycogen to lactic acid, glycolytic muscles are more sensitive to postmortem glycolysis [53]. This results in a lower pH, which exerts a cascade of metabolic processes that instigate increased cooking loss and reduced sarcomere length, muscle fiber size, inosine, hypoxanthine and IMP. Further, the negative relationships with %fat and SFA suggest that glycolytic muscles have a low %fat and SFA. This was contrary to oxidative or oxido-glycolytic muscles that are known to have high intramuscular fat, which implies that fat oxidation is necessary to supply the high energy requirement for cellular metabolisms [54,55]. The second axis, PC2, is more related to the muscle fiber type, as it is positively loaded by MyHCIIb and negatively loaded by MyHCI and MyHCIIx. MyHCIIb is closely related to cGPD, MUFA and GMP, in contrast to MyHCI and MyHCIIx which are closely related to A-FABP, pH_45_, pH_24_, a* and shear force. This finding highlights the typical diversity in muscle fibers in relation to their physicochemical properties. The results of the bi-plot and the effects of GR on a few of the variables that were shown in Table 3, Table 4 and Table 5 had comparable trends. This draws attention to specific attributes of the loin of different TNPs. Remarkably, a clear separation in meat quality between the NT loin and other TNPs was observed. In line with our results, PCA demonstrated distinct variations in the meat quality of several pig breeds [56].

## 5. Conclusions

The findings of this study demonstrate that TNPs from various geographical regions had comparable levels of MyHC expression, metabolic-related gene expression and IMF content. In comparison to the other loins, the NT loin had lower ribonucleotides, a lower sarcomere length and higher levels of cooking loss, PUFA and glycogen. These results provide valuable insights for optimizing the utilization of Thai native pork and highlight the importance of considering regional variations in meat quality parameters. Further studies examining the impact of age, sex and slaughter weight on the muscle fiber composition and meat quality in TNPs are recommended.

## Figures and Tables

**Table 1:** List of primers.

Gene ^1^		Primer Sequence (5′ to 3′)	Annealing Temperature, °C	Accession No.	Reference
MyHC I	Forward:	AAGGGCTTGAACGAGGAGTAGA	60	AB053226	[11]
	Reverse:	TTATTCTGCTTCCTCCAAAGGG			
MyHC IIA	Forward:	GCTGAGCGAGCTGAAATCC	60	AB025260	
	Reverse:	ACTGAGACACCAGAGCTTCT			
MyHC IIX	Forward:	AGAAGATCAACTGAGTGAACT	60	AB025262	
	Reverse:	AGAGCTGAGAAACTAACGTG			
MyHC IIB	Forward:	ATGAAGAGGAACCACATTA	57	AB025261	
	Reverse:	TTATTGCCTCAGTAGCTTG			
A-FABP	Forward:	TACTGAGATTGCCTTCAAATTGGG	60	-	[19]
	Reverse:	TCTGGTAGCCGTGACACCTTTC			
TPI−1	Forward:	GCCAAATAATAAGCCACATCCA	56	gi|194042634	[16]
	Reverse:	AGGCGACACCATCAGAAGCA			
PGAM−1	Forward:	GATGTGGTACGTCCCTCTGC	60	gi|374694	
	Reverse:	GGCACTTACAGGCGTATTCAG			
cGPD	Forward:	TGTGATGGGCTGGGCTTTG	60	gi|2149958	
	Reverse:	GTGATGAGGTCGGCGATGC			
GAPDH	Forward:	TCACTGCCACCCAGAAGA	65	ABO38240	[22]
	Reverse:	TACCAGGAAATGAGCTTGAC			

^1^ Adipocyte fatty acid binding protein (A-FABP), triose phosphate isomerase 1 (TPI−1), phosphoglycerate mutase 1 (PGAM−1), cytosolic glycerol-3-phosphate dehydrogenase (cGPD) and glyceraldehyde-3-phosphate dehydrogenase (GAPDH).

**Table 2:** Expression of myosin heavy chain isoforms, fat deposition and metabolism-related genes in *Longissimus thoracis* of Thai native pigs from different geographical regions.

Gene ^1^	Geographical Region (GR)			RMSE	*p* Value
	NT	ST	NE		
MyHC I ^2^	0.53	0.24	0.55	0.63	0.473
MyHC IIa ^2^	3.41	8.92	3.79	5.17	0.058
MyHC IIx ^2^	71.90	67.86	73.27	10.87	0.522
MyHC IIb ^2^	24.14	23.37	22.37	8.79	0.894
A-FABP ^3^	1.04	1.10	1.51	0.71	0.271
TPI−1 ^3^	1.05	0.82	0.72	0.37	0.163
PGAM−1 ^3^	1.42	1.11	1.00	0.73	0.407
cGPD ^3^	1.50	1.75	1.38	0.85	0.631

^1^ Adipocyte fatty acid binding protein (A-FABP), triose phosphate isomerase 1 (TPI−1), phosphoglycerate mutase 1 (PGAM−1) and cytosolic glycerol-3-phosphate dehydrogenase (cGPD). ^2^ Relative expression ratio. ^3^ Gene expression of the target gene: GAPDH. NT = Northern Thai native pigs, ST = Southern Thai native pigs, NE = Northeastern Thai native pigs. RMSE = Root mean square error.

**Table 3:** Chemical composition, glycogen and ribonucleotide contents in *Longissimus thoracis* of Thai native pigs from different geometric regions.

Trait	Geographical Region (GR)			RMSE	*p* Value
	NT	ST	NE		
Fat (%)	3.07	3.55	3.35	1.13	0.575
Moisture (%)	71.98	70.97	71.40	1.48	0.221
Glycogen (mg/g wet weight)	55.22 ^a^	28.12 ^b^	18.22 ^b^	13.17	<0.0001
Ribonucleotides (mg/100 g)					
Hypoxanthine	2.03 ^b^	5.46 ^a^	4.31 ^a^	2.09	0.004
Inosine	15.10 ^b^	55.30 ^a^	41.17 ^a^	16.81	0.0001
Inosine monophosphate	144.43 ^b^	284.98 ^a^	277.37 ^a^	77.07	0.003
Guanosine monophosphate	2.41	2.65	2.16	1.24	0.748

^a,b^ Means with different superscripts in a row are significantly different (*p* < 0.05). NT = Northern Thai native pigs, ST = Southern Thai native pigs, NE = Northeastern Thai native pigs, RMSE = Root mean square error.

**Table 4:** Meat characteristics of *Longissimus thoracis* of Thai native pigs from different geographical regions.

Trait	Geographical Region (GR)			RMSE	*p* Value
	NT	ST	NE		
pH_45_	6.09	6.18	6.30	0.34	0.264
pH_24_	5.48 ^b^	5.56 ^ab^	5.66 ^a^	0.12	0.002
L*	56.65	57.70	55.09	1.84	0.055
a*	5.71 ^a^	4.21 ^b^	6.22 ^a^	1.52	0.012
b*	14.83	15.06	14.22	2.34	0.666
Cooking loss (%)	20.79 ^a^	14.87 ^b^	14.86 ^b^	3.06	<0.0001
Shear force (kg)	5.67	5.04	5.74	1.31	0.374
Muscle fiber diameter (µ)	48.63	53.28	53.51	7.24	0.131
Sarcomere length (µ)	1.83 ^b^	1.95 ^a^	1.93 ^a^	0.09	0.002

^a,b^ Means with different superscripts in a row are significantly different (*p* < 0.05). pH_45_ = measured at 45 min postmortem. pH_24_ = measured at 24 h postmortem. NT = Northern Thai native pigs, ST = Southern Thai native pigs, NE = Northeastern Thai native pigs, RMSE = Root mean square error.

**Table 5:** Fatty acid composition (% of total fatty acid) of *Longissimus thoracis* in Thai native pigs from different geographical regions.

Fatty Acid	Geographical Region (GR)			RMSE	*p* Value
	NT	ST	NE		
C14:0	1.83	1.64	1.69	0.37	0.466
C16:0	17.22 ^b^	20.89 ^ab^	23.39 ^a^	4.39	0.009
C16:1	9.43	8.14	7.62	2.12	0.126
C18:0	9.63	10.87	11.61	3.73	0.436
C18:1n9c	39.82	45.44	42.24	6.58	0.154
C18:2n6c	17.14 ^a^	9.17 ^b^	9.63 ^b^	4.87	0.001
C18:3n3	0.47	0.47	0.65	0.40	0.454
C20:4n6	3.04 ^a^	1.44 ^b^	1.69 ^b^	0.84	0.0003
C20:1	1.02	1.56	1.15	0.61	0.118
C20:2	0.40	0.36	0.31	0.19	0.539
∑n-3	0.47	0.47	0.65	0.40	0.454
∑n-6	20.18 ^a^	10.61	11.32	3.21	0.002
SFA	28.68 ^b^	33.41 ^a^	36.70 ^a^	5.17	0.004
MUFA	50.27	55.14	51.01	7.00	0.236
PUFA	21.04 ^a^	11.45 ^b^	12.29 ^b^	6.08	0.002
P:S	0.75 ^a^	0.34 ^b^	0.34 ^b^	0.24	0.0004
n-6/n-3	42.93 ^a^	22.57 ^b^	17.41 ^b^	6.23	0.005
Atherogenicity index	0.30 ^b^	0.41 ^a^	0.47 ^a^	0.06	0.003
Thrombogenicity index	0.77 ^b^	0.97 ^a^	1.10 ^a^	0.09	0.001

^a,b^ Means with different superscripts in a row are significantly different (*p* < 0.05). NT = Northern Thai native pigs, ST = Southern Thai native pigs, NE = Northeastern Thai native pigs, RMSE = Root mean square error. Total monounsaturated fatty acids, ∑MUFA = C16:1 + C18:1n9c + C22:1; total polyunsaturated fatty acids, ∑PUFA = C18:2n6c + C18:3n3 + C20:2n + C20:4n6; total saturated fatty acids, ∑SFA = C14:0 + C16:0 + C18:0; P:S = ∑PUFA ÷ ∑SFA. ∑n6 = C18:2n-6 + C20:4n-6; ∑n3 = C18:3n-3. Atherogenicity index = (C12:0 + 4 × C14:0 + C16:0)/(ΣMUFA + Σn6 + Σn3). Thrombogenicity index = (C14:0 + C16:0 + C18:0)/[(0.5 × ΣMUFA + 0.5 × Σn6 + 3 × Σn3) + (n3/n6. SFA (saturated fatty acids): C14:0 + C16:0 + C18:0; MUFA (monounsaturated fatty acids): C16:1 + C18:1n9c + C20:1; PUFA (polyunsaturated fatty acids): C18:2n6c + C20:4n6 + C20:2 + C18:3n3.

## Data Availability

The original contributions presented in the study are included in the article, further inquiries can be directed to the corresponding author.
